# Resting Heart Rate and Outcomes in Patients with Cardiovascular Disease: Where Do We Currently Stand?

**DOI:** 10.1111/j.1755-5922.2012.00321.x

**Published:** 2013-07-18

**Authors:** Ian BA Menown, Simon Davies, Sandeep Gupta, Paul R Kalra, Chim C Lang, Chris Morley, Sandosh Padmanabhan

**Affiliations:** 1 Craigavon Cardiac CentreCraigavon, UK; 2 Royal Brompton HospitalLondon, UK; 3 Whipps Cross and St Bartholomew’s HospitalsLondon, UK; 4 Portsmouth Hospitals NHS TrustPortsmouth, UK; 5 Ninewells Hospital and Medical SchoolDundee, UK; 6 Bradford Royal InfirmaryBradford, UK; 7 BHF Glasgow Cardiovascular Research Centre, University of GlasgowGlasgow, UK

**Keywords:** Angina, Beta-blockers, Calcium channel blockers, Cardiovascular risk, Coronary artery disease, Heart failure, If Channel blockers

## Abstract

**Background:**

Data from large epidemiological studies suggest that elevated heart rate is independently associated with cardiovascular and all-cause mortality in patients with hypertension and in those with established cardiovascular disease. Clinical trial findings also suggest that the favorable effects of beta-blockers and other heart rate–lowering agents in patients with acute myocardial infarction and congestive heart failure may be, at least in part, due to their heart rate–lowering effects. Contemporary clinical outcome prediction models such as the Global Registry of Acute Coronary Events (GRACE) score include admission heart rate as an independent risk factor.

**Aims:**

This article critically reviews the key epidemiology concerning heart rate and cardiovascular risk, potential mechanisms through which an elevated resting heart rate may be disadvantageous and evaluates clinical trial outcomes associated with pharmacological reduction in resting heart rate.

**Conclusions:**

Prospective randomised data from patients with significant coronary heart disease or heart failure suggest that intervention to reduce heart rate in those with a resting heart rate >70 bpm may reduce cardiovascular risk. Given the established observational data and randomised trial evidence, it now appears appropriate to include reduction of elevated resting heart rate by lifestyle +/− pharmacological therapy as part of a secondary prevention strategy in patients with cardiovascular disease.

## Introduction

Epidemiological data over the last six decades have found that elevated resting heart rate (RHR) may be associated with increased risk of all-cause mortality and cardiovascular (CV) mortality, both in the general population (with or without risk factors) 1, and in those with established CV disease 2–4. Clinical trial findings in patients with established CV disease suggest that pharmacological reduction of elevated RHR may be associated with improved outcomes. RHR is included in clinical risk prediction models such as the Global Registry of Acute Coronary Events (GRACE) score for patients with acute coronary syndrome, but its role as an independent risk factor and therapeutic target for the management of CV disease is less clear 5. The aim of this article is to critically review the evidence regarding the prognostic implications of elevated RHR in patients with hypertension and established CV disease, the mechanisms through which an elevated RHR may be disadvantageous, and how reduction in elevated RHR may be of potential benefit.

## Prognostic Role of Heart Rate in Hypertensive Individuals

An early description of the association between elevated RHR and mortality in hypertension was described in the Framingham Study dataset. During 36-year follow-up of those with any blood pressure >140/90 mmHg but not on antihypertensive treatment (n = 4530), the adjusted odds of all-cause mortality for a RHR increment of 40 bpm was 1.98 for men and 1.87 for women 6. While the association between RHR and mortality in hypertensive women has been less clear in other studies 7 and likely begins at a higher RHR compared with men 8, as with recent general population studies 1, the weight of evidence in hypertension suggests that elevated RHR is an important risk factor for mortality in both sexes. In the French Centre d’Investigations Préventives et Cliniques (IPC) study (n = 19 386), RHR was significantly associated with all-cause mortality in both men and women. In men, RHR independently predicted CV mortality (*P* < 0.001). In women (in whom mortality rates were generally low), RHR >100 bpm versus <60 bpm was associated with a modest increase in CV mortality that was not significant (3.3% vs. 2.0%; *P* = 0.53) but a trend to increase stroke mortality (2.0% vs. 0.6%; *P* = 0.054) 7. In the Systolic Hypertension in Europe (Syst-Eur) study conducted in elderly patients (mean age, 70 years) with systolic hypertension, RHR >79 bpm was a significant predictor of all-cause, CV, and non-CV mortality 9. In the Losartan (vs. atenolol) Intervention For Endpoint reduction in hypertension (LIFE) study 10, multivariate analysis found every 10 bpm increase in RHR was associated with a 16% increased risk of CV mortality and 25% increased risk of all-cause mortality, independent of blood pressure lowering, randomized treatment assignment or other risk factors including left ventricular hypertrophy.

In the Anglo-Scandinavian Cardiac Outcomes Trial (ASCOT) 11 and Valsartan Antihypertensive Long-term Use Evaluation (VALUE) 12 studies, follow-up accumulated mean levels of RHR were better predictors of CV events than baseline RHR. In the Glasgow Blood Pressure Clinic Study 13, hypertensive patients with persistently elevated RHR (>80 bpm) had an increased risk of all-cause and CV mortality. Of interest, the highest risk of all-cause mortality was seen in those patients who increased their RHR by ≥5 bpm by the end of follow-up (1.51 [95% CI, 1.03–2.20]; *P* = 0.035). The LIFE study 10 also reported that persistence or development of a RHR ≥84 bpm during follow-up was associated with an 89% increased risk of CV mortality and a 97% increased risk of all-cause mortality. Such data indicate that in addition to baseline RHR, change in RHR during treatment is also an important predictor of long-term survival.

## Heart Rate and Outcomes in Established Cardiovascular Disease

### Acute Coronary Syndromes

Increased RHR in patients with acute myocardial infarction (MI) is an important predictor of subsequent death, independent of the presence of heart failure, hypotension/shock, and other clinical variables. In the pre-thrombolytic era, Hjalmarson et al. 14 showed, in 1807 patients with acute MI, that RHR on admission independently predicted 1-year mortality. Among 1044 patients hospitalized for acute MI in the Secondary Prevention Reinfarction Israeli Nifedipine Trial-2 (SPRINT-2), a 15 bpm increase in admission RHR independently predicted increased in-hospital mortality (hazard ratio, 1.36; 95% confidence interval [CI], 1.08–1.72) and 1-year mortality (hazard ratio, 1.45[95% CI, 1.15–1.84]) 15.

In the thrombolytic era, among 11 267 post-MI patients in the Gruppo Italiano per lo Studio della Sopravvivenza nell’Infarto miocardico-3(GISSI-3) trial, admission RHR 81–100 bpm compared with RHR <60 bpm was associated with double the in-hospital mortality (6.3% vs. 3.3%) and discharge RHR 81–100 bpm was associated with fourfold higher mortality at 6 months (9.3% vs. 1.9%) 16. In the Global Utilization of Streptokinase and Tissue plasminogen activator for Occluded coronary arteries-1 (GUSTO-1) trial (n = 41 021), a model containing admission RHR plus four other independent variables predicted approximately 90% of 30-day deaths 17. In the GISSI-Prevenzione study (n = 11 324 patients with recent MI [<3 months]), RHR at discharge or on follow-up assessment of ≥75 bpm, particularly in men, was an independent predictor of 4-year mortality 18.

In 9461 patients with non-ST elevation acute coronary syndrome in the Platelet glycoprotein IIb/IIIa in Unstable angina: Receptor Suppression Using Integrilin (eptifibatide) Therapy (PURSUIT) trial, admission RHR (median, 72 bpm; interquartile range, 63–80 bpm) was the second strongest predictor of 30-day mortality 19. In those with non-ST elevation MI, admission RHR of 80 versus 72 bpm was associated with a 28% excess adjusted risk of 30-day mortality. Serial reports from the GRACE study in acute coronary syndrome (with or without ST elevation) 20 reported admission RHR (per 30 bpm increase) to be an independent predictor of death, in-hospital and at 6 months. The GRACE risk prediction tool, developed from a cohort of 43 810 patients (21 688 derivation set; 22 122 validation set), is based on admission RHR, in addition to age, systolic blood pressure, Killip class, cardiac arrest at admission, serum creatinine, and elevated cardiac markers 20. A strength of the GRACE registry, in contrast to randomized clinical trials, is its recruitment of all-comers and thus potentially broader applicability to an unselected coronary care population. Discharge or follow-up RHR data such as that from GISSI-3 16, GISSI-Prevenzione 18, and GRACE 20 are of particular interest as they are less likely to be affected by acute clinical factors (which may or may not have been fully accounted for during multivariate analysis).

In 1453 patients undergoing primary angioplasty for ST elevation MI, Antoni et al. reported that discharge heart rate ≥70 bpm (vs. <70 bpm) was independently associated with a twofold increase in risk of CV mortality at 1- and 4-year follow-up 21.

**Figure 1:**
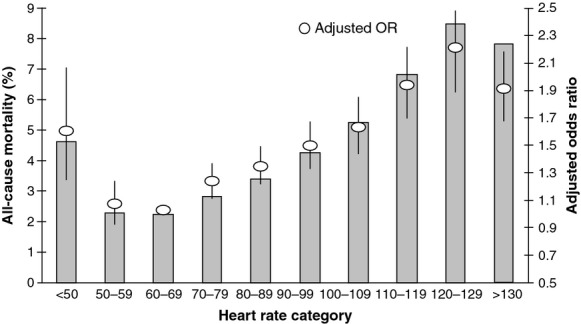
Relationship between presenting HR and all-cause mortality (absolute percentage and adjusted odds ratio compared with a nadir of 60–69 bpm) from 135 164 patients with non-ST elevation acute coronary syndrome in the CRUSADE registry study. Reproduced with permission from Bangalore et al. 22.

Registry data from 135 164 non-ST-elevation acute coronary syndrome patients enrolled in CRUSADE (Can Rapid risk stratification of Unstable angina patients Suppress ADverse outcomes with Early implementation of the American College of Cardiology/American Heart Association Guidelines) 22 found that presentation heart rate of 90–99 bpm versus 60–69 bpm (adjusted for baseline confounders and excluding those with shock) was associated with an increased primary composite outcome of death, re-MI and stroke (OR, 1.21 [95%CI,1.10–1.32]), and increased all-cause mortality (OR, 1.49 [95%CI, 1.32–1.68]). This association remained consistent whether or not beta-blockers were used. Interestingly, a J-shaped curve was noted with risk increasing below a presentation heart rate nadir of 50 bpm (Figure 1). This contrasts with studies from general 1 or hypertensive 6 populations 1.

### Stable Coronary Disease

Heart rate may also be a prognostic factor in patients with stable coronary heart disease (CHD). Diaz et al. 2 reported long-term data from 24 913 patients in the landmark Coronary Artery Surgery Study (CASS) registry (median, 14.7 years follow-up). Increasing RHR was associated with higher rates of all-cause mortality, CV mortality, and CV rehospitalization (*P* < 0.0001). A RHR of ≥83 bpm, compared with <62 bpm, was associated with 32% increase in total mortality and 31% increase in CV mortality even after adjustment for multiple potentially confounding clinical variables.

Frank et al. 23 reported that in patients 2 months after nonurgent coronary artery bypass surgery, ambulatory heart rate >90 bpm versus <60 bpm was independently associated with risk of CV events during long-term follow-up (hazard ratio, 2.26 [95% CI, 1.04–4.91]; *P* = 0.04), with a trend to all-cause mortality (hazard ratio, 3.57 [95% CI, 0.90–14.17]; *P* = 0.07).

In the Angina and Silent Ischaemia Study (ASIS), a rise in RHR of at least 5 bpm preceded 81% of ischemic episodes recorded during 48-h ambulatory ECG monitoring 24. Patients with RHR >80 bpm were twice as likely to develop ischemia as those with rates <60 bpm (16.6% vs. 8.7%, respectively).

The importance of baseline and on-treatment RHR was evaluated in 22 576 patients with stable CHD plus hypertension enrolled in the INternational VErapamil-SR/trandolapril STudy (INVEST), 3 assigned to a rate lowering antihypertensive strategy of either atenolol (twice daily) or verapamil. Elevated baseline RHR showed a modest linear association with risk of death, MI, or stroke from 55 to 100 bpm, with a sharp increase in risk at >100 bpm (approximately twofold vs. RHR < 100 bpm). In contrast, elevated RHR at follow-up (on-treatment) was strongly associated with increased risk, even at intermediate levels. An increase in follow-up RHR from 70 bpm to 80 bpm was associated with 31% excess risk. Of note, baseline RHR did not retain significance when assessed in a multivariate analysis including follow-up RHR, suggesting that follow-up (on-treatment) RHR is a more important predictor of future adverse events. Similar patterns were seen for males, females, and those with prior MI or diabetes. As with the CRUSADE registry, a J-shaped curve was noted with risk increasing below a RHR nadir of 59 bpm. Low RHR did not appear to be caused by excessive treatment dose; the mean atenolol dose was less; and the mean verapamil dose was unchanged in such patients. Adverse outcomes were similar in the atenolol and verapamil groups.

In the placebo arm of the large randomized BEAUTIfUL (morBidity-mortality EvAlUaTion of the I_f_ inhibitor ivabradine in patients with coronary disease and left ventricULar dysfunction) trial, 4 patients with RHR ≥70 bpm (n = 2693) versus RHR <70 bpm (n = 2745) had an increased adjusted incidence of CV death (34%; *P* = 0.0041), MI (46%; *P* = 0.0066), coronary revascularization (38%; *P* = 0.037), or admission with heart failure (53%; *P* < 0.0001). Each 5 bpm increment in RHR was associated with approximately 8% increase in CV death, 7% increase in MI, 8% increase in coronary revascularization, and 16% increase in admission with heart failure (although of note, the increase in death and heart failure outcomes rose continuously ≥70 bpm, whereas the relationship was less pronounced for MI and revascularization).

Rambihar et al. 25 reported from a population of 31 531 patients with stable CV disease that the highest versus lowest mean RHR quintile (>78 bpm vs. ≤58 bpm) was associated with a 77% increase in CV mortality and 65% increase in all-cause mortality.

### Heart Failure

Resting heart rate is also an independent predictor of mortality in patients with heart failure. In the placebo arm of Metoprolol controlled release/Extended Release randomized Intervention Trial in Heart Failure (MERIT-HF) 26, RHR was a highly significant independent risk factor for all-cause mortality (*P* = 0.003), CV mortality (*P* = 0.006), and worsening heart failure (*P* < 0.0001). Those with RHR in the highest (>90 bpm) versus lower 4 quintiles had 51% increased all-cause mortality, 90% increased heart failure mortality, and 78% increased hospitalization for worsening heart failure. In the Cardiac Insufficiency BIsoprolol Study-II (CIBIS-II), which assessed bisoprolol in 2647 patients with symptomatic chronic heart failure, baseline RHR independently predicted mortality and hospitalization for worsening heart failure 27. In a meta-analysis of patients treated with carvedilol, bisoprolol, metoprolol, bucindolol, or nebivolol 28, final achieved RHR correlated strongly with all-cause mortality (*P* < 0.005; nine trials, n = 19 537) and the amount of change in RHR correlated with change in ejection fraction (*P* < 0.005; 26 trials, n = 3389). While some of the benefit of beta-blockers maybe attributed to prevention of dysrhythmias, reduction of free fatty acid use in favor of glucose and reduced cardiac myocyte apoptosis, RHR reduction appeared the predominant mechanism of benefit 28. In the placebo arm of the Systolic Heart failure treatment with the I_f_ inhibitor ivabradine Trial (SHIFT), patients with RHR in the highest versus lowest quintile (≥87 bpm vs. 70 to <72 bpm) were over twice as likely to have a CV death or hospitalization for worsening heart failure (HR, 2.34; 95% CI, 1.84–2.98; *P* < 0.0001), the risk increasing by 16% for every 5 bpm increase in RHR 29.

## Pathophysiological Mechanisms

The precise pathophysiological mechanisms that link RHR and CV outcomes are not fully defined. However, this link appears independent of lifestyle and other conditions that may contribute to elevated RHR 1, such as alcohol consumption, advancing age, blood pressure, anemia, diabetes, obesity, and reduced levels of physical activity. Because pre-existing or subclinical CV disease may lead to elevations in RHR, reverse causality could influence the relationship between RHR and the future development of CV disease. Some studies have aimed to minimize this possibility by excluding events within the first 1–2 years from analysis; the demonstration of a persistent relationship thereby supporting a temporal sequence consistent with causality 1. RHR is determined by sinus node activity, which is largely influenced by the interaction of sympathetic and vagal activity. Therefore, an elevated RHR may reflect sympathovagal imbalance resulting from sympathetic overactivity or a decrease in vagal activity. Both of these may increase the risk of life-threatening arrhythmic events and sudden death in myocardial ischemia and in heart failure. Sympathovagal imbalance may also be associated with inflammation and thus potentially lead to atherosclerosis. Sajadieh et al. 30 found an inverse association between RHR, heart rate variability and C-reactive protein (CRP) in healthy individuals, and that a combination of CRP and heart rate variability or RHR was predictive of subsequent death or MI. Rogowski et al. 31 studied 4553 apparently healthy men and reported that individuals in the top versus bottom quintile of RHR (>79 bpm vs. <58 bpm) had significant increases in fibrinogen, high-sensitivity CRP, and the absolute number of polymorphonuclear leukocytes.

**Figure 2:**
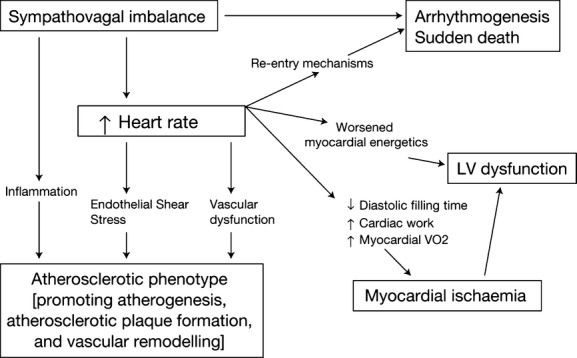
Biological mechanisms linking heart rate and cardiovascular outcomes.

Elevated RHR per se may have direct adverse impact on CV risk and CV function (Figure 2). Altered myocardial energetics may impair myocardial efficiency. Heart rate is related to vascular elastance; hence, ventricular loading 32. Heart rate reduction thus unloads the ventricle, the effect being greatest in diseased hearts. Myocardial ischemia may result from an increase in myocardial oxygen consumption and reduced diastolic filling time, which reduces coronary blood flow. Elevated RHR is associated with arterial stiffness 33 and with abnormal (low and oscillatory) patterns of endothelial shear stress 34. Abnormal shear stress may activate endothelial mechanoreceptors (including ion channels, G-proteins, tyrosine kinase receptors, nicotinamide adenine dinucleotide phosphate [NADPH], and xanthine oxidase), in turn triggering a complex network of several intracellular pathways (known as mechanotransduction), and thereby leading to an atherogenic endothelial phenotype that promotes atherogenesis and risk of plaque rupture 34. Elevated RHR is also associated with increase in blood pressure-derived circumferential tensile stress that, via mechanoreceptor and mechanotransduction processes, may alter the endothelial phenotype to a pro-atherosclerotic state 34. Heidland and Strauer found that RHR >80 bpm was an independent predictor of new plaque rupture in patients undergoing a second angiogram within 6 months of an original angiogram 35.

More detailed examination of pathophysiological mechanisms including ambulatory assessment is discussed elsewhere 1,9,34 but collectively, these findings support the hypothesis that RHR, through several hemodynamic and nonhemodynamic mechanisms, may directly influence CV outcome.

## Is Pharmacological Reduction in Heart Rate of Clinical Value?

While epidemiological and preclinical data support the hypothesis that elevated RHR is a risk marker for adverse CV outcome, the clinical value (or otherwise) and the mechanisms of benefit of intervention to lower elevated RHR is a separate and more challenging question because the benefits of some heart rate–lowering agents may be mediated via nonheart rate mechanisms.

### Beta-Blockers

Multiple trials have shown that beta-blockers improve outcomes in patients with acute MI 36. Potential mechanisms of benefit include an anti-ischemic action (strongly associated with heart rate reduction) and also an anti-arrhythmic action. Analysis of 8 trials in which early intravenous beta-blocker therapy was given found a near-linear association between RHR and infarct size (r = 0.97; *P* < 0.001), a ≥15 bpm RHR reduction being associated with 25–30% reduction in infarct size 36. Analysis of 11 long-term, placebo-controlled beta-blocker trials following acute MI found RHR strongly associated with mortality (r = 0.60; *P* < 0.05) and recurrent MI (r = 0.59; *P* < 0.05), benefits being most marked in those with higher RHR. In contrast, beta-blockers with intrinsic sympathomimetic activity showed limited reduction in RHR and no significant effect on mortality 36.

The Euro Heart survey of stable angina suggested that use of beta-blockers may be suboptimal. Although beta-blockers were the most commonly added treatment, mean doses used were relatively low compared with clinical trials. While a high baseline heart rate (>83 bpm) was more often noted in patients with chronic respiratory disease (23.1%) and diabetes (22.6%), beta-blockers were less likely to be added in these patients 37.

In heart failure, the CIBIS-II study (in which bisoprolol, compared with placebo, was associated with 34% reduction in all-cause mortality and 44% reduction in sudden death), patients with the lowest baseline RHR or greatest decrease in RHR during treatment had the lowest mortality and lowest risk of readmission. A reduction of >10 bpm was associated with the best chance of survival, provided systolic blood pressure did not fall excessively 27 (in which case further RHR lowering is likely of hemodynamic disadvantage^38^).

It is noteworthy that in CIBIS-II, patients with no change in RHR still derived some benefit from bisoprolol treatment, 27 and, in MERIT-HF, the reduction in mortality and hospitalization with extended-release metoprolol versus placebo appeared independent of baseline RHR and magnitude of RHR reduction, suggesting that RHR reduction was not the sole mechanism of benefit 26. Other beta-blocker benefits in heart failure potentially include improved cardiac myocyte metabolism, reduction in apoptosis, reduction in atrial and ventricular arrhythmias, and reduction in myocardial ischemia and/or hibernation.

Nevertheless, in a small but intriguing study of 49 pacemaker-dependent patients with heart failure, reversal of the apparent benefit of the beta-blocker-induced bradycardia on left ventricular volume and systolic function occurred in those randomized to a high-pacing rate (80 bpm) versus those paced at 60 bpm, supporting RHR reduction as an important mechanism by which beta-blockers improve heart failure outcomes 39.

By contrast, in the setting of hypertension, despite observational studies showing the typical association between elevated RHR and adverse outcome, the value of beta-blockers appears lower than might be expected. The ASCOT trial 11, which was conducted in 19 257 hypertensive patients (without prior MI) and stopped early after a median of 5.5 years, found that, compared with an atenolol ± bendroflumethiazide-based strategy, an amlodipine ± perindopril-based strategy was associated with a trend to a lower incidence of the primary endpoint of nonfatal MI and fatal coronary heart disease (4.5% vs. 4.9%; *P* = 0.1052), with significant reductions in secondary endpoints, including all-cause mortality (8% vs. 9%; *P* < 0.025), total CV events and procedures (14% vs. 17%; *P* < 0.0001), fatal and nonfatal stroke (3% vs. 4%; *P* = 0.0003), and incidence of new-onset diabetes (6% vs. 8%; *P* < 0.0001). However, the authors noted that approximately half of the difference in outcome may be accounted for by a lower on-treatment blood pressure in the amlodipine-based arm (maximum 5.9/2.4 mmHg at 3 months; mean, 2.7/1.9 mmHg) and emergence in the atenolol arm of higher BMI, fasting glucose, creatinine, triglycerides, and lower HDL-cholesterol 40. In-trial heart rate at 6 weeks, in contrast to baseline heart rate, was a predictor of adverse events. In addition, the value of atenolol versus other beta-blockers in hypertension has been questioned, given its low lipophilicity, disadvantageous effect on central aortic blood pressure 41 and tendency to increase insulin resistance (potentially exacerbated in ASCOT by concurrent thiazide use). Nevertheless, in the absence of prior MI, beta-blockers are not currently recommended for first-line antihypertensive therapy.

### Calcium Channel Blockers

Nondihydropyridine calcium channel blockers, which lower heart rate, have been shown to reduce the risk of cardiac events in post-MI patients 42. TheDAnish Verapamil Infarction Trial II (DAVIT II) randomized 1775 patients in week 2 post-MI (mean RHR 75 bpm) to verapamil 120 mg tid or placebo. Beta-blockers were an exclusion criteria. Compared with placebo, verapamil, which reduced mean pulse by 6 bpm and blood pressure by 5/4 mmHg, showed a trend to reduce mortality at 18 months (hazard ratio, 0.80; *P* = 0.11), and a significant reduction in the composite of cardiac death/MI (hazard ratio, 0.80; *P* = 0.03) 42. Of note, in those without heart failure (n = 1161), verapamil was associated with a significant reduction in both 18-month mortality (7.7 versus 11.8%; hazard ratio, 0.64; *P* = 0.02) and reinfarction (9.4% vs. 12.7%; hazard ratio, 0.67; *P* = 0.02). By contrast, in those with heart failure (n = 614), verapamil showed a slight, nonsignificant excess in death (17.9% vs. 17.5%) and reinfarction (14.3% vs. 14.2%). The Multicenter Diltiazem Post-Infarction Trial (MDPIT) randomized 2466 patients with acute MI (mean RHR, 71 bpm) to diltiazem (60 mg qid) or placebo, for 12–52 months. Over half were also receiving beta-blockers 43. Overall, diltiazem was associated with no difference in mortality, and only a small, nonsignificant reduction in death/MI (16% vs. 18%; hazard ratio 0.9 [95% CI 0.74–1.08]). As with verapamil, diltiazem was associated with reduced cardiac death/MI, compared with placebo, in those without pulmonary congestion (n = 1909; hazard ratio, 0.77 [95%CI, 0.61–0.98]). However, in those with pulmonary congestion (n = 490), diltiazem was associated with a significant increase in cardiac death/MI (hazard ratio, 1.41 [95% CI, 1.01–1.96]). A similar increased risk was present for patients with an ejection fraction below 40% 43. Thus, while verapamil or diltiazem may be beneficial in post-MI patients without heart failure, in contrast to beta-blockers, they are typically discouraged in patients with heart failure or impaired left ventricular function.

An important recent meta-regression analysis of 17 randomized controlled studies (14 with beta-blockers and three with calcium channel blockers) assessed whether improvements in outcome following MI were related to RHR reduction 44. RHR reduction was significantly associated with reduction in cardiac death (*P* < 0.001), all-cause death (*P* = 0.008), sudden death (*P* = 0.015), and nonfatal reinfarction (*P* = 0.024). Larger RHR reduction was associated with greater improvement in outcome—each 10 bpm reduction in RHR reduced the risk of cardiac death by approximately 30%. Heterogeneity analysis found outcome differences between trials were fully explained by achieved RHR reduction, rather than drug class. In particular, the absence of mortality reduction with calcium channel blockers appeared entirely because of their minimal reduction in RHR.

### I_f_ Channel Blockers

Several ion channels or currents control sino-atrial node pacemaker activity, including the I_f_ channel. This channel is an inward-mixed sodium and potassium current, directly regulated by intracellular cyclic adenosine monophosphate (excitatory) and muscarinic (inhibitory) receptors. The I_f_ current is a key determinant of the slope and duration of diastolic depolarization and thus of overall heart rate. Early I_f_ channel inhibitors were too nonselective to be of clinical value. However, the highly selective I_f_ channel blocker ivabradine has good tolerability, with a mean reduction in RHR of 8–10 bpm at usual doses and similar anti-ischemic benefits to atenolol 45, but without reduction in blood pressure or cardiac contractility and thus represents an intriguing agent to test the heart rate hypothesis.

**Figure 3:**
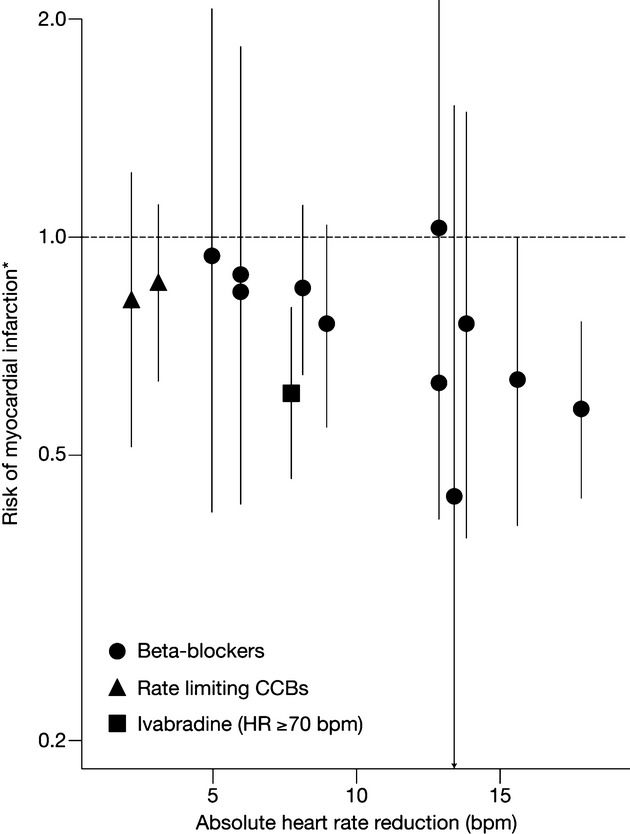
Reduction in risk of myocardial infarction and reduction in resting heart rate by beta-blockers, calcium channel blockers (adapted with permission from Cucherat et al. [44]) and ivabradine (data from [4]). *Odds ratio data for beta-blockers and calcium channel blockers; hazard ratio data for ivabradine.

In BEAUTIfUL, 10 917 patients with CHD and left ventricular ejection fraction <40% were randomized to ivabradine (5–7.5 mg bid) or placebo, for a median of 19 months 46. Most (87%) were already taking beta-blockers, and mean baseline RHR was already relatively low (71.6 bpm). At study doses used, ivabradine versus placebo reduced overall mean RHR by just 6 bpm which was not associated with reduction in the primary composite endpoint of CV death or hospitalization for acute MI or heart failure (15.4% vs. 15.3%; hazard ratio, 1.0 [95% CI, 0.91–1.10]). In prespecified secondary analysis of those with initial RHR ≥70 bpm (mean, 79 bpm; n = 5392), ivabradine did not reduce the primary composite endpoint, but was associated with consistent reduction in multiple coronary endpoints including hospitalization for fatal/nonfatal MI (3.1% vs. 4.9%; hazard ratio, 0.64 [95% CI, 0.49–0.84]) and coronary revascularization (2.8 vs. 4.0; hazard ratio, 0.70 [95% CI, 0.52–0.93])—the degree of coronary event reduction in keeping with that predicted by the Cucherat meta-regression (Figure 3). Post hoc analysis was thus undertaken in those with limiting angina (n = 1507; 13.8%) 47 which reported ivabradine to be associated with a borderline significant 24% reduction in the primary endpoint (hazard ratio, 0.76 [95% CI, 0.58–1.00]; *P* = 0.05) and a 42% reduction in hospitalization for MI (hazard ratio, 0.58 [95% CI, 0.37–0.92]). Further reductions in coronary endpoints were seen in the subgroup with limiting angina plus RHR ≥70 bpm (n = 712) albeit with wide confidence intervals due to the smaller subgroup numbers. Given the neutral primary endpoint in BEAUTIfUL, such coronary endpoint reductions can only be considered as hypothesis generating, and thus, a prospective clinical trial is currently being undertaken (SIGNIfY—Study assessing the mortality/morbidity benefits of the I_f_inhibitor ivabradine in patients with coronary artery disease).

**Figure 4:**
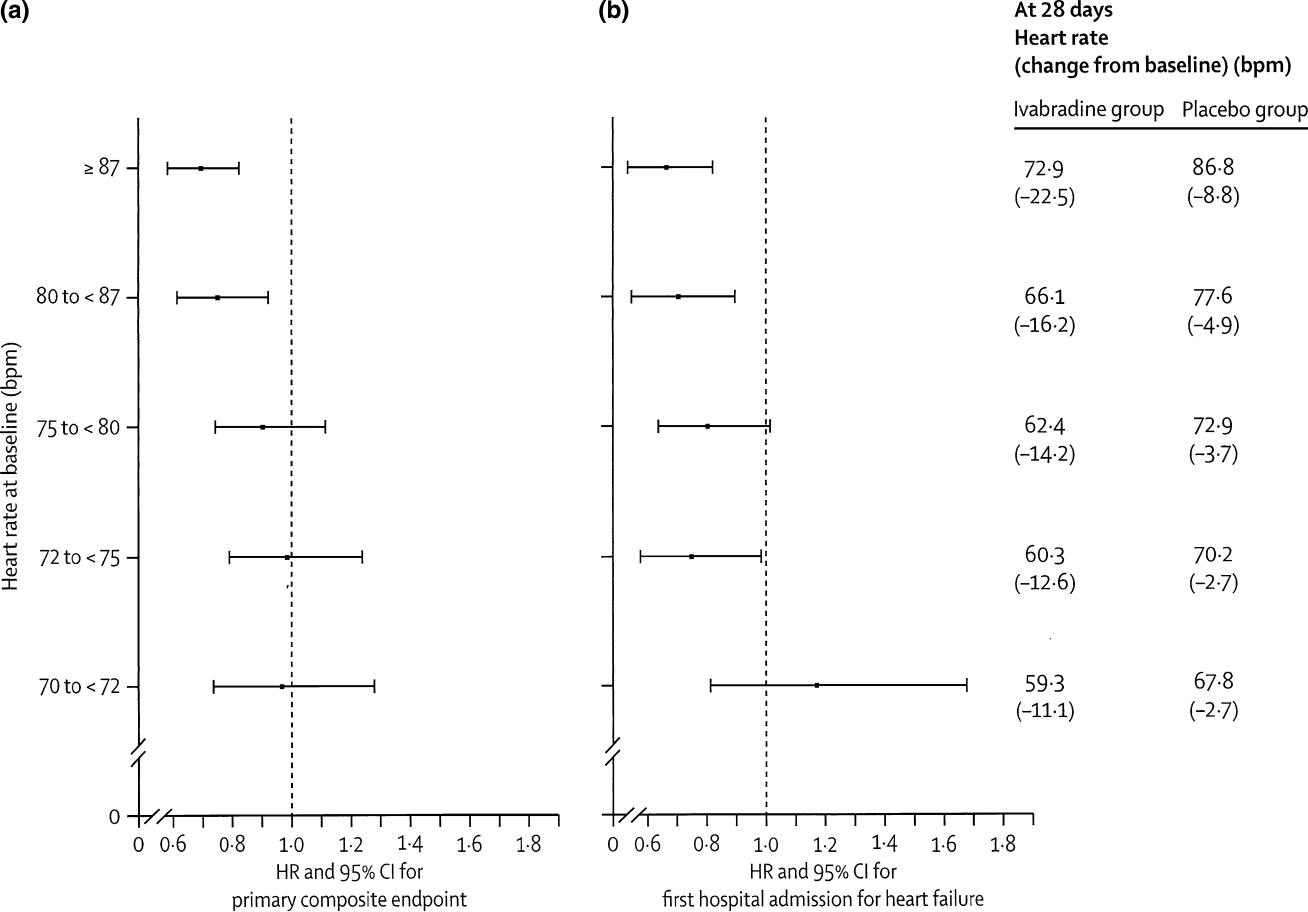
Effect of ivabradine compared with placebo on the primary composite endpoint and first hospital admissions for worsening heart failure by quintiles of baseline heart rate distribution (adapted with permission from Böhm et al. 29).

Most recently, SHIFT 48 randomized 6505 patients with stable symptomatic heart failure (NYHA Class II to IV), EF ≤35%, resting heart rate >70 bpm and hospitalization for heart failure in the previous 12 months to ivabradine 7.5 mg twice daily or placebo on top of standard medication including maximally tolerated beta-blockade. Only half of patients were able to achieve >50% of target beta-blockade dose. At median follow-up (22.9 months), the primary composite endpoint of CV death or hospital admission for worsening heart failure was significantly reduced by ivabradine group (24% vs. 29%; hazard ratio, 0·82 [95% CI, 0·75–0·90]; *P* < 0·0001), mainly driven by a reduction in hospital admission for heart failure and deaths caused by heart failure. Event rates were lowest in those achieving a 28-day RHR <60 bpm. The event reduction with ivabradine was greatest in patients with highest baseline heart rates (Figure 4) 29. This was noted by the European Medicines Agency that specified a baseline heart rate of ≥75 bpm in ivabradine’s recent heart failure licence. While previous studies have confirmed elevated RHR to be a risk marker, the SHIFT data suggest elevated RHR may be a risk factor—the treatment benefit of ivabradine (mechanistically, a heart lowering agent without other hemodynamic or neurohormonal benefit) being neutralized if adjusted for change of heart rate at 28 days) 29.

### Digoxin

Digoxin reduces RHR mainly by enhancing parasympathetic activity nervous system although it probably also inhibits the sympathetic nervous system. Its use in heart failure declined after its failure to reduce all-cause mortality in the Digitalis Investigation Group (DIG) Trial 49. However, a recent retrospective analysis of DIG 50, that assessed the primary composite endpoint used in SHIFT (i.e., CV death or hospital admission for worsening heart failure) reported a similar risk reduction in the composite outcome (hazard ratio, 0.85; 95% CI, [0.79–0.91]; *P* < 0.001) driven, like SHIFT, by a significant reduction in heart failure hospitalization (hazard ratio, 0.72; 95% CI, [0.66–0.79]; *P* < 0.001). Of note, patients in DIG were not treated with a beta-blocker and such a combination can infrequently lead to atrioventricular block. However, unlike ivabradine, digoxin is beneficial in the setting of atrial fibrillation. While prospective data are needed 51, there would appear to be merit in a reappraisal of digoxin’s role in heart failure.

### Other Agents

A number of new specific heart rate–lowering agents with potential value for treatment of angina are currently in development, including YM 758 (another I_f_ channel inhibitor) 52 and Bay-68-4986 (an oral adenosine A1 receptor agonist) 53.

## Conclusion

Multiple studies, in those with established CV disease, with CV risk factors, and even apparently healthy subjects, have shown elevated RHR is a marker of increased risk. Increased risk has been well described in many studies for RHR >80 bpm, with newer data demonstrating increased risk >70 bpm. In the setting of coronary artery disease 3, 22, a J-shaped curve has been noted with very low RHR <50 bpm appearing counterproductive. Of clinical interest are randomized data from patients with significant coronary heart disease or heart failure suggesting that intervention to reduce elevated RHR rate reduces CV risk. Given the established observational data and randomized recent trial evidence, it now appears appropriate to aim to reduce elevated RHR (by lifestyle ± pharmacological therapy) as part of a secondary prevention strategy in patients with CV disease.
